# Duck Enteritis Virus VP16 Antagonizes IFN-*β*-Mediated Antiviral Innate Immunity

**DOI:** 10.1155/2020/9630452

**Published:** 2020-05-15

**Authors:** Yang Li, Mingshu Wang, Anchun Cheng, Renyong Jia, Qiao Yang, Shun Chen, Dekang Zhu, Mafeng Liu, Xinxin Zhao, Shaqiu Zhang, Juan Huang, Xumin Ou, Sai Mao, Yanling Yu, Ling Zhang, Yunya Liu, Leichang Pan, Bin Tian, Mujeeb Ur Rehman, Xiaoyue Chen

**Affiliations:** ^1^Institute of Preventive Veterinary Medicine, Sichuan Agricultural University, Wenjiang 611130, China; ^2^Avian Disease Research Center, College of Veterinary Medicine of Sichuan Agricultural University, Wenjiang 611130, China; ^3^Key Laboratory of Animal Disease and Human Health of Sichuan Province, Sichuan Agricultural University, Wenjiang 611130, China

## Abstract

Duck enteritis virus (DEV) can successfully evade the host innate immune responses and establish a lifelong latent infection in the infected host. However, the study about how DEV escapes host innate immunity is still deficient up to now. In this study, for the first time, we identified a viral protein VP16 by which DEV can obviously downregulate the production of IFN-*β* in duck embryo fibroblast (DEF). Our results showed that ectopic expression of VP16 decreased duck IFN-*β* (duIFN-*β*) promoter activation and significantly inhibited the mRNA transcription of IFN-*β*. Further study showed that VP16 can also obviously inhibit the mRNA transcription of interferon-stimulated genes (ISGs), such as myxovirus resistance protein (Mx) and interferon-induced oligoadenylate synthetase-like (OASL). Furthermore, we found that this anti-interferon activity of VP16 depended on its N-terminus (aa1-200). Coexpression analysis revealed that VP16 selectively blocked duIFN-*β* promoter activity at the duIRF7 level rather than duIRF1. Based on the results of coimmunoprecipitation analysis (co-IP) and indirect immunofluorescence assay (IFA), VP16 was able to bind to duck IRF7 (duIRF7) directly, but did not interact with duck IRF1 (duIRF1) in vitro.

## 1. Introduction

Duck enteritis virus (DEV) is a kind of enveloped, large DNA virus who belongs to *α*-herpesvirus. Most of the research about DEV mainly focus on etiology [1–8], pathogenesis [9–13], epidemiology, and diagnosis [14–23]. All of the waterfowls are apt to suffer from this disease which could cause considerable mortality and morbidity [24–26]. As a prevalent pathogen spreading in waterfowl, it causes acute fever and septic disease in some cases. But in the chronic cases, DEV contributes to latent infections in the TG (trigeminal ganglia) after establishing a primary infection. Using TG as a base, reactivation of the virus may lead to disease outbreaks [24]. In common with other members of *α*-herpesvirus family, DEV possesses the ability to establish latency in the sensory ganglia and lymphoid tissues [27]. The innate immune system plays an essential role in defending the host against viral infection. They could detect the presence of pathogens and initiate relevant mechanisms to eliminate potentially infectious threats. This protective process will occur following the intruding nucleic acid recognition initiated by germline-encoded molecules termed PRRs (pattern recognition receptors) [28, 29], which could trigger a range of signaling cascades and subsequent host gene activation [30]. PRRs recognize conserved features of viruses and other microorganisms. They were investigated and known as PAMPs (pathogen-associated molecular patterns), which include microbial nucleic acids, proteins, and carbohydrates. PAMPs own key molecular characteristics shared by classes of pathogens are usually absent from healthy cells [31].

In the case of double-stranded DNA viruses like DEV, the situation of immunization reacting is a little more complicated than those of RNA viruses, partly because their constitute elements are the same with host DNAs and the deficiency of triphosphorylated groups in their 5′ ends, which RIG-I and MDA5 utilize to distinct microbial RNAs from host messenger RNAs [32–34]. Up to now, three major receptors that initiate DNA-driven immune responses have been identified, including TLR9 (Toll-like receptor 9), absent in AIM2 (melanoma 2), and cGAS (cyclic GMP-AMP synthase) [35]. Moreover, some proteins, including DAI, RNA polymerase III, IFI16, DDX41, and others like DNA helicases, have been suggested to function as the potential DNA sensors that induce interferon, even though the details of their mechanisms remain to be elusive [36–38].

Interferon type I and type II are important components of the host antiviral innate immune system which both induce ISG (IFN-stimulated gene) expression through JAK- (Janus kinase-) dependent phosphorylation of signal transducer and activator of transcription STAT1 and STAT2. They have strong and broad-spectrum antiviral effects, so they are also known as “antiviral interferon.” Among birds, interferons of chickens and ducks are the most studied [39, 40]. IFN-*β*, which belongs to the interferon type I family, is a primarily immunosuppressive factor to inhibit virus clearance during viral infection [41], whereby promoting the occurrence of chronic viral infection [42]. At the same time, IFN (interferon) has a strong inhibitory effect on a variety of viruses by inducing the synthesis of antiviral proteins or promoting the function of immune cells. Therefore, the ability of virus to escape or antagonize IFN reaction to a certain extent determines the fate of virus and host in the future. Almost all the viruses, both DNA virus and RNA virus, have developed multifarious strategies to interfere with the synthesis of IFNs or IFN receptor signaling pathway, including reducing the expression of type I IFN receptor mRNA, blocking the posttranslation modification, degrading the IFN receptor, and utilizing the virus bait protein to bind to type I interferon to prevent the receptor from recognizing interferon and inhibiting JAK/STAT signal, further regulating the antiviral function of ISG products [43, 44].

The characteristics of some genes from DEV had been reported [45–64]. However, the function of VP16 of duck enteritis virus encoded by the UL48 gene was hardly studied. In contrast to DEV, the function of another tegument protein VP16 from HSV-1 was well elucidated [65–68]. The transcriptional expression of IFN-*β* is regulated by the transcriptional enhancer bound to its promoter, which includes the regulatory region of IRF3/IRF7, AP-1 (activating protein 1), and the regulatory region of NF-*κ*B. Intriguingly, in spite of IRF3 deficiency in chicken, poultries may employ IRF7 to reconstitute corresponding IFN signaling to respond to both DNA and RNA viral infections [69]. Therefore, in this study, for the first time we identified that VP16 could directly interact with duck IRF7 whereby the VP16 of DEV plays a vital role in antagonizing the innate immune system, assisting DEV virus to counteract this antiviral activity.

## 2. Materials and Methods

### 2.1. Virus, Cells, and Reagents

The DEV strain was provided by the Institute of Preventive Veterinary Medicine, Sichuan Agricultural University [70]. The virus titer was measured as the 50% tissue culture infective dose (TCID50) according to previously described methods [71]. Duck embryo fibroblasts (DEFs) used in this study were isolated from 10-day-old duck embryo. And the DEF was cultured in minimum essential medium (MEM) supplemented with 10% newborn calf serum (Gibco) and incubated at 37°C with 5% CO_2_ in an incubator. HEK 293T cells (ATCC) were grown in Dulbecco's modified Eagle medium (DMEM, Gibco) containing 10% fetal bovine serum (FBS) and incubated at 37°C in a 5% CO_2_ humidified cabinet the same as DEF. Poly (I:C) (pIC) and poly (dA:dT) were purchased from InvivoGen (San Diego, USA).

### 2.2. RNA Isolation and cDNA Preparation

Total RNA was isolated using RNAiso Plus Reagent (TaKaRa) according to the manufacturer's instructions. Genomic DNA was then removed, and reverse transcription was performed using a PrimeScript™ RT Reagent Kit (Perfect Real Time, TaKaRa) according to the manufacturer's instructions.

### 2.3. Plasmids

To construct pCAGGS-VP16-HA, pCAGGS-VP16 (aa1-200)-HA, pCAGGS-VP16 (aa1-390)-HA, and pCAGGS-duIRF1-Flag plasmids, DEV UL48 (GenBank: NC_013036) [72] and duck IRF1 (GenBank: NC_006127) sequences were amplified from cDNA extracted from DEV-infected DEFs with PCR primers (Table 1) and were integrated into the pCAGGS vector using a one-step cloning kit (Vazyme). Results and conclusion of sequence analysis demonstrated that recombinant plasmids have sequences in line with data from GenBank. pCAGGS-duIRF7-Flag, pCAGGS-duSTING-Flag, pCAGGS-duTBK1-Flag, pCAGGS-ducGAS-Flag, and duIFN-*β*-Luc were kindly provided by Chen. pRL-TK as internal control plasmid was purchased from Promega.

### 2.4. Subcellular Localization

The procedure was performed as described previously with slight modification [73]. When the HEK293T cells seeded on coverslips grew into 90% confluence, plasmids pCAGGS-UL48-HA and pCAGGS-duIRF7-Flag were cotransfected using TransIn™ EL Transfection Reagent. 48 hours after transfection, discarding the cell culture and washing the cells with PBST (phosphate-buffered saline with Tween 20) three times, the cells were fixed in albumin in PBST for 24 hours at 4°C and incubated with rabbit-anti-HA or mouse-anti-Flag for 2 hours at 37°C. Following washing with PBST three times, the cells were then incubated with secondary antibodies, namely, Alexa Fluor 488 Goat anti-rabbit and Alexa Fluor 568 Goat anti-mouse, both of which diluted in PBST.

### 2.5. Western Blot, Coimmunoprecipitation

The procedure was performed as described previously with slight modification [74]. The DEV VP16 was expressed efficiently in duck embryo fibroblasts (DEFs) and human embryonic kidney (HEK) 293T cells. For western blot detection, cells were lysed in RIPA lysis buffer (Beyotime, China) with 1 mM phenyl methane sulfonyl fluoride (PMSF) for 40 minutes at around 4°C. Lysed cells were centrifuged at 14000 revolutions per minute (RPM) for 5 minutes and were combined with 10x sodium dodecyl sulfate (SDS) loading buffer, boiled for 10 minutes at 100°C, and separated by SDS-polyacrylamide gel electrophoresis (SDS-PAGE). Afterwards, the separated proteins were electroblotted onto polyvinylidene difluoride (PVDF) membranes (Millipore, USA) and subjected to immunoblotting with a primary antibody against Flag (CST, America), HA (CST, America), or VP16 (Sichuan Agricultural University), a secondary antibody against mouse or rabbit. For coimmunoprecipitation, protein of interest was well expressed in HEK293T cells and revolved as above. Before SDS-PAGE, we added corresponding primary antibody (MBL, America) up to 1 *μ*g/*μ*L, incubating in a 4°C refrigerator, fixing on a turntable for about 8 hours. Then, 1/10 volume of lysed cells of protein A+G (Beyotime, China) mingled in this processed cellular samples, incubating for another 5 hours at the same circumstance as above. After washing three times with phosphate-buffered saline (PBS) supplemented with Tween (PBST), the samples were boiled in 10x sodium dodecyl sulfate (SDS) loading buffer by SDS-PAGE.

### 2.6. Dual-Luciferase Reporter Assay

DEFs made from 10-day duck embryo were seeded in 24-well plates as described previously [74, 75]. Cells were cotransfected using Lipofectamine 3000 (Invitrogen) according to the manufacturer's instructions with 500 ng/well of the specific expression plasmids or an empty vector together with 500 ng/well reporter plasmid IFN-*β*-Luc until cells grow into 70~80% density, as well as 50 ng/well of the pRL-TK as an internal control vector (Promega). At 24 h post transfection, cells were treated or not treated with 25 *μ*g/mL poly (I:C) or poly (dA:dT). These cells were stimulated with poly (I:C) or poly (dA:dT) for over 24 h and harvested by phosphate-buffered saline (PBS). Firefly luciferase activity was measured by the dual-luciferase assay system (Promega) according to the manufacturer's directions; then, corresponding data were processed by GraphPad Prism 7.00.

### 2.7. Real-Time Quantitative PCR

The procedure was performed as described previously with slight modification [76]. Briefly, total cellular RNA was extracted using RNAiso Plus Reagent (TaKaRa) according to the manufacturer's instructions. Then, the RNA was reverse transcribed into cDNA by means of PrimeScript™ RT Reagent Kit. The threshold cycle (Ct) values were normalized to the housekeeping gene *β*-actin. The relative expression levels of IFN-*β*, Mx, and OASL were determined using *β*-actin as an endogenous control as well as comparative Ct (−2^*ΔΔ*in^) method and a real-time thermocycler (CFX96 Bio-Rad, Hercules, CA, USA). Real-time qPCR primers were designed as Table 1.

### 2.8. Statistical Analysis

Student's *t*-test was used to evaluate the differences. *P* values < 0.05 were considered significant.

## 3. Results

### 3.1. DEV Could Inhibit the Activation of Duck IFN-*β* Promoter through the cGAS-STING-Mediated DNA Sensing Pathway as well as at the IRF Level

**Figure 1:**
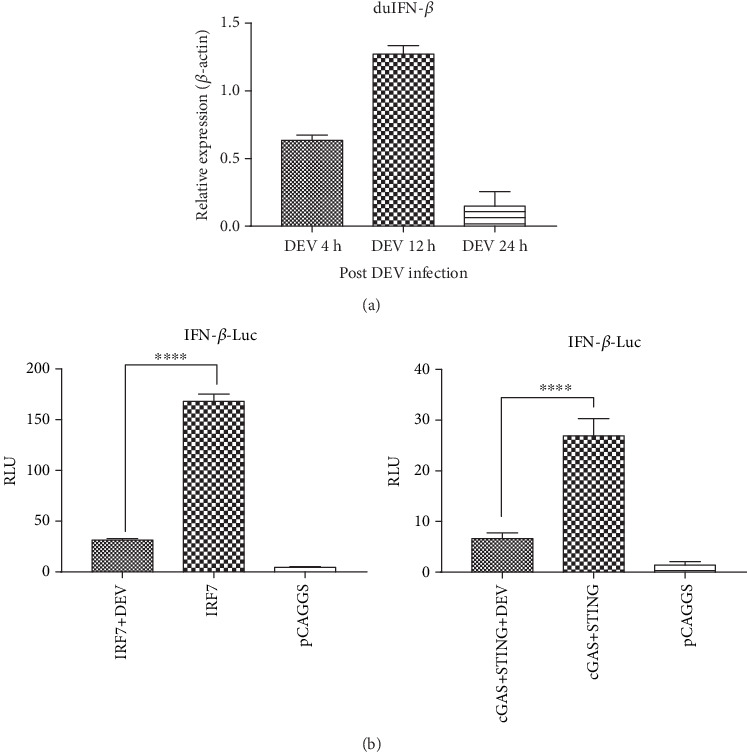
The immune evasion of DEV in vitro. (a) DEFs were cultured in 6-well plates and infected with WT DEV (1 MOI) when they grew up to 90%. At 4 hours, 12 hours, and 24 hours, the infected cells were collected and real-time qPCR was performed to determine the transcriptional levels of duck IFN-*β* in the DEF. (b) DEFs were seeded in 24-well plates and transfected with 500 ng of the duIFN-*β*-Luc (duck IFN-*β* promoter reporter plasmid), together with 50 ng of pRL-TK (Renilla luciferase plasmid, Promega, America) and pCAGGS empty vector or plasmids encoding the indicated protein (duck IRF7, duck STING, or duck cGAS, 500 ng/well). Cells were mock infected or infected with 0.5 MOI WT DEV 12 hours posttransfection, and firefly luciferase activities were measured at 48 h postinfection; the data were analyzed by GraphPad Prism software, and results were presented using two-way ANOVA (*n* = 3) and considered significant (^∗∗∗∗^*P* < 0.0001).

To explore the effect of DEV infection on IFN-*β* production in vitro, DEFs (duck embryo fibroblasts) were inoculated with DEV (duck enteritis virus). They were infected with DEV for 4, 12, and 24 hours, respectively, and then duck IFN-*β* mRNA levels were analyzed by rt-qPCR (real-time quantitative PCR). The DEFs exhibited slight responses at 12 hours postinfection, while that became lower at 24 hours (Figure 1(a)). Therefore, we put forward a hypothesis that the DNA sensing pathway was able to recognize DEV, but the reaction may be suppressed during the later phase of DEV replication. In consideration of cGAS acting as the most effective cytosolic exogenous DNA sensing in most DNA viruses [77], I ventured a guess whether DEV is involved in the cGAS-STING-mediated pathway. By using Dual-Luciferase Reporter Gene System, I found that DEV could inhibit duck IFN-*β* promoter activity that was upregulated by ectopic expression of duck cGAS and STING (Figure 1(b)). In response to cellular stimulation, activation of IRF7 is achieved by phosphorylation of IRF7. Upon phosphorylation from TBK1 activated by STING, IRF7 dimerizes and translocates to the nuclei, acting as a transcription factor. The dipolymer complex of IRF7 can then bind to the IRF7 receptor sites within the IFN-*β* promoter region, eventually activating the transcription of the IFN-*β* gene [69]. Therefore, I verified whether DEV could inhibit IRF7-stimulated IFN-*β* promoter activity. The results showed that DEV was able to diminish IFN-*β* promoter activity at IRF7 level (Figure 1(c)).

### 3.2. DEV VP16 Repressed Activation of duIFN-*β* and Interferon-Stimulated Genes (ISGs) Induced by Poly (I:C) or Poly (dA:dT)

**Figure 2:**
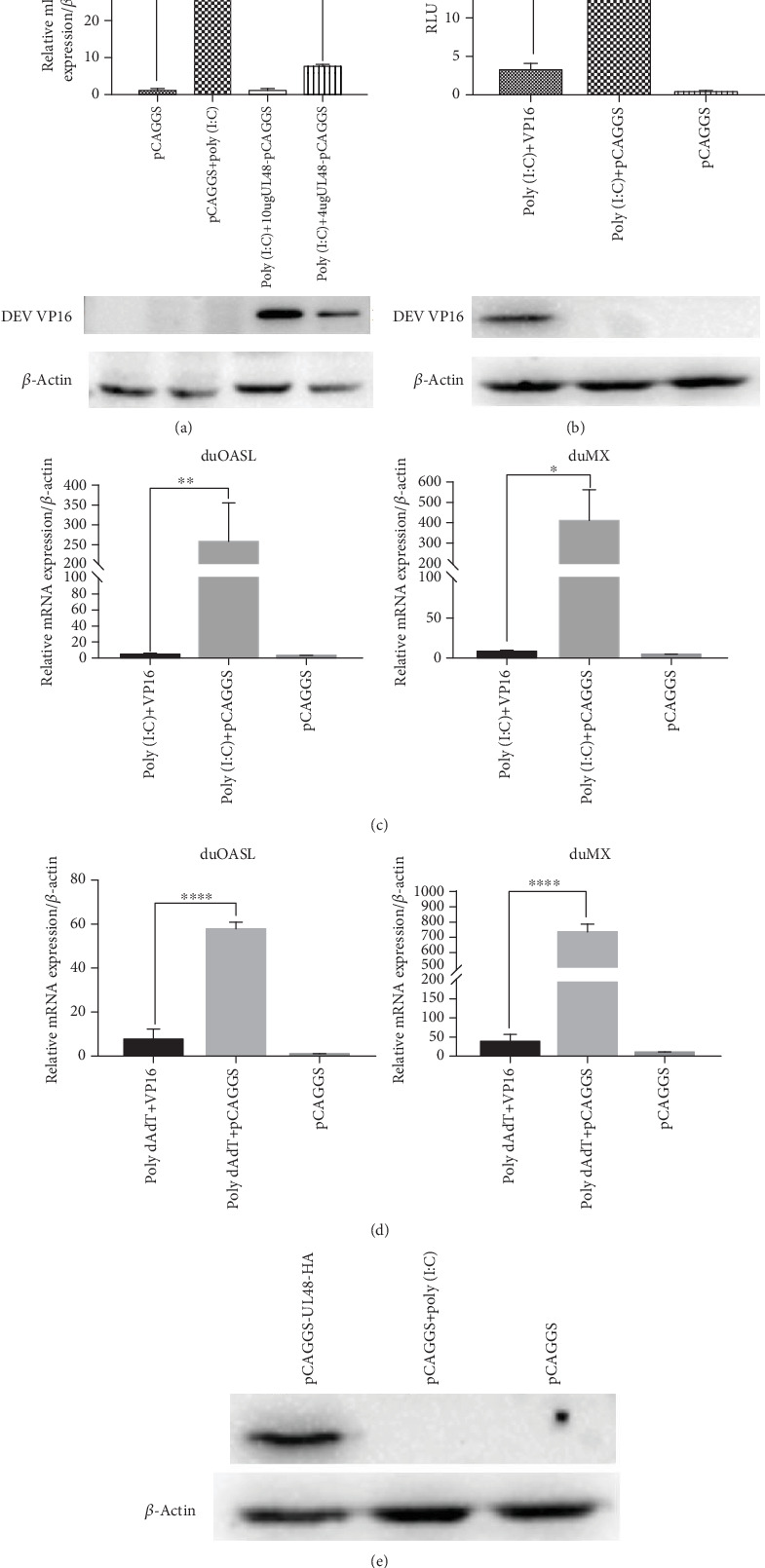
DEV VP16 is having a demonstrable effect on IFN-*β* activation and IFN-*β*-related ISGs. (a) Duck IFN-*β* mRNA relative levels in DEF transfected 4 *μ*g or 10 *μ*g pCAGGS-UL48-HA, stimulated with 25 *μ*g/mL poly (I:C) for over 36 hours at 12 hours posttransfection, were presented by real-time qPCR. The expression of VP16 was analyzed by western blotting using anti-HA and anti-*β*-actin (as a control) (CST, America). In this group, an increased amount of VP16-HA expression plasmid was used. (b) DEFs seeded in 24-well plates were transfected with pCAGGS or pCAGGS-UL48-HA (500 ng/well), together with duIFN-*β*-Luc (500 ng/well) and pRL-TK (50 ng/well) for 12 hours; then, 25 *μ*g/mL poly (I:C) was transfected into cells for another 24 hours. Firefly luciferase activities were measured and the data were analyzed by GraphPad Prism software, and results were presented using two-way ANOVA (*n* = 3). (c, d) Quantitative real-time PCR analysis of duck OASL and duck Mx mRNA levels in DEF cells which express VP16-HA or not. Duck ISG mRNA relative expression was upregulated by (c) poly (I:C) or (d) poly (dA:dT). Experiments were shown using two-way ANOVA (*n* = 3). Significant, ^∗^*P* < 0.05, ^∗∗^*P* < 0.01, and ^∗∗∗∗^*P* < 0.0001. (e) Expression of VP16-HA was confirmed by western blot analysis.

To test whether DEV VP16 could repress activation of duIFN-*β*, rt-qPCR was performed to measure mRNA accumulation of duIFN-*β*, duMx, duOASL. As shown in Figures 2(a) and 2(c), the mRNA levels of duIFN-*β*, duMx, and duOASL induced by poly (I:C)—a double-stranded RNA (dsRNA) analog—were significantly higher than those transfected with pCAGGS-UL48-HA. Moreover, the mRNA levels of duMx and duOASL activated by poly (dA:dT)—a double-stranded DNA analog—were downregulated, too (Figure 2(d)). At the same time, the expression of VP16 was identified by western blot (Figure 2(e)). Additionally, VP16 inhibited IFN-*β* mRNA transcription in a dose-dependent manner (Figure 2(a)); the expression of VP16 protein was confirmed by western blot analysis (Figure 2(a)). The data from Dual-Luciferase Reporter Gene System also showed that ectopic expression of VP16 significantly inhibited poly (I:C)-induced activation of the IFN-*β* promoter (Figure 2(b)), similar to the results of rt-qPCR.

### 3.3. DEV VP16 Could Inhibit cGAS-STING-Mediated Pathway at the IRF7 Level rather than IRF1

**Figure 3:**
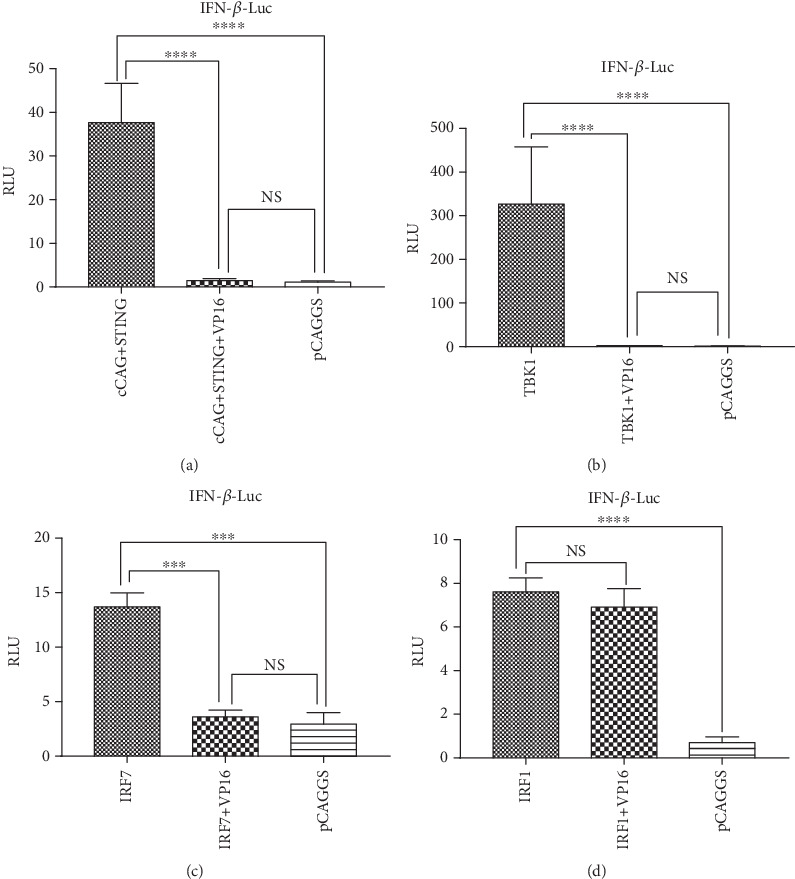
DEV VP16 could inhibit IFN-*β* promoter activity at IRF level but not IRF1. DEFs were cultured in 24-well plates and then cotransfected with duIFN-*β*-Luc (500 ng), pRL-TK (50 ng), and appointed plasmids encoding (a) STING and cGAS, (b) TBK1, (c) IRF7, and (d) IRF1. Luciferase assays were performed as described for Figure 1 to measure the activation of the IFN-*β* promoter.

**Figure 4:**
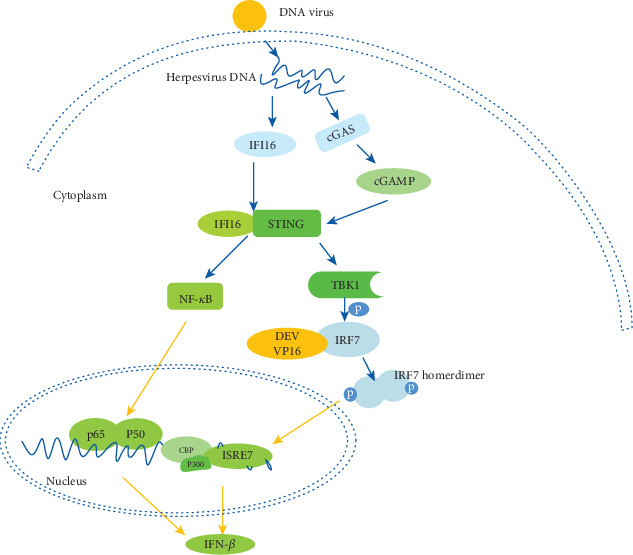
A sketch of DNA sensing pathway mainly induced by herpesvirus and how the VP16 of DEV exerts function in immune evasion strategy.

In order to identify where VP16 protein hinders IFN-*β* production during the cGAS-STING pathway, we utilized different stimuli to induce the IFN-*β* promoter activity in DEFs. In the past research, duck STING and IRF7 were confirmed to have the capacity to induce IFN-*β* production pathway [78, 79]. We overexpressed duck STING, cGAS, TBK1, IRF7, and IRF1 in DEFs and analyzed the activity of IFN-*β* promoter reporter in the presence of VP16 protein or not. As a result, ectopic expression of cGAS and STING significantly stimulated the IFN-*β* promoter activity in DEF while this activation was suppressed by the existence of VP16 (Figure 3(a)). Similarly, when we transfected the downstream immunologic factors including TBK1, IRF7, IFN-*β* promoter activity was upregulated and VP16 could also inhibit their activation ability for IFN-*β* promoter (Figures 3(b) and 3(c)). Interestingly, the duck IRF1 was able to activate IFN-*β* promoter activity as other counterparts did, but its function was not dampened by the overexpression of VP16 (Figure 3(d)). A sketch map of IFN-*β* pathway mediated by cGAS is shown (Figure 4).

### 3.4. DEV VP16 Associated Directly with Duck IRF7 to Block the Activation of Duck IFN-*β* but Did Not Interact with Duck IRF1

**Figure 5:**
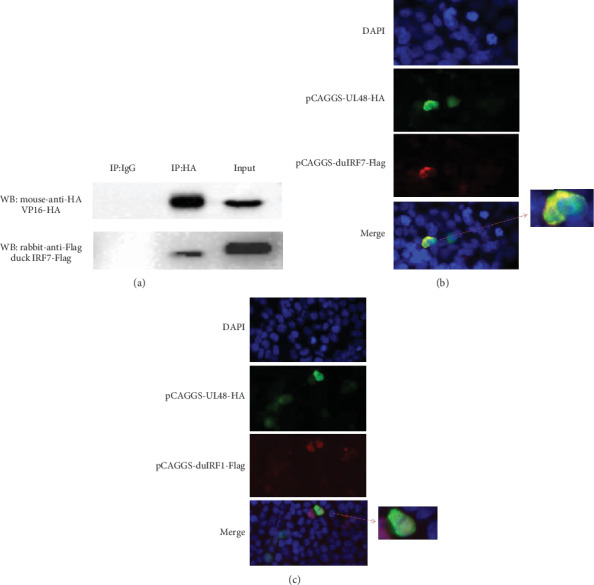
DEV VP16 associates with duck IRF7 but not with duck IRF1. HEK293T cells were cotransfected with pCAGGS-UL48-HA and pCAGGS-IRF7-Flag or pCAGGS-IRF1-Flag. (a) At 48 hours after transfection, cells were resolved to coimmunoprecipitation and immunoblot analysis with the indicated antibodies. (b) Colocalization of UL48 (green) and IRF7 (red) in HEK293T cells, the nuclei were stained by DAPI (1 : 1000) (blue). (c) Localization of UL48 (green) and IRF1 (red) in HEK293T cells. Nuclei stained by DAPI were shown in blue.

The transcription of IFN-*β* can be dependent on the activation of IRF7 [78]. It may take the place of IRF3 in poultry to bind to distinct regulatory domains in the IFN-*β* promoter. To clarify what are the mechanisms when IFN-*β* is suppressed by DEV VP16 in vitro, contrasting the result of Figure 3(d) with the obvious inhibition result of IFN-*β* promoter activity induced by IRF7 (Figure 3(c)) promoted us to investigate the possibility of an interaction between these two proteins. HEK-293T cells were transfected with VP16-HA along with IRF7-Flag, and coimmunoprecipitation assay was performed with anti-hemagglutinin (anti-HA) and anti-Flag antibodies. We found that IRF7 protein was immunoprecipitated by VP16 (Figure 5(a)). To further verify the interaction between duck IRF7 and DEV VP16, we exerted immunofluorescence colocalization analysis, which revealed colocalization of IRF7 and VP16 proteins in HEK293T cells (Figure 5(b)). Meanwhile, the immunofluorescence subcellular colocalization analysis between IFR1 and VP16 revealed the IRF1 could not interact with VP16 in vitro (Figure 5(c)).

### 3.5. The N-Terminal of VP16 Is Responsible for Inhibiting Poly (I:C)-Mediated Activation of duIFN-*β* Promoter

**Figure 6:**
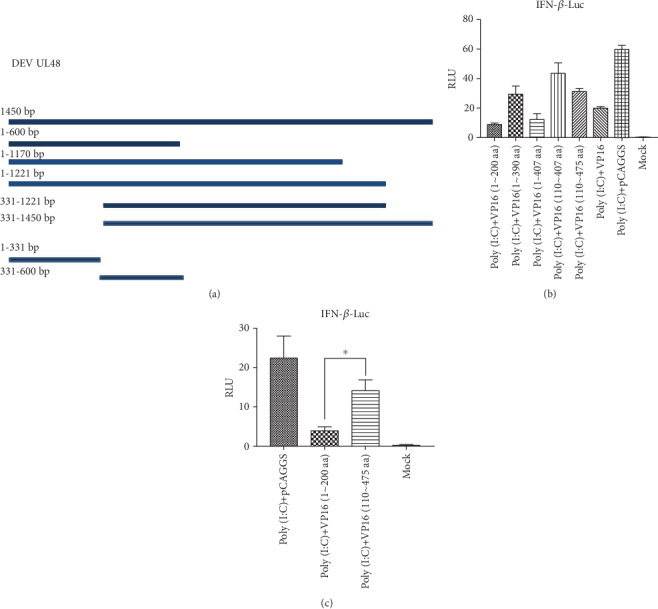
N-terminal of VP16 plays the most effective role in the inhibition of IFN-*β* promoter activity. (a) Construction of different kinds of truncated VP16 protein. (b, c) Transfection of DEFs that were cultured in 24-well plates with duIFN-*β*-Luc (500 ng), pRL-TK (50 ng), and VP16 or plasmids encoding truncated VP16, the same with that described in Figure 2(a).

In order to further find the regions of VP16 that are involved in its inhibition in IFN-*β* pathway, we constructed a series of plasmids with truncated mutants of VP16, including VP16 (aa1-200), VP16 (aa1-390), VP16 (aa1-407), VP16 (aa110-407), and VP16 (aa110-475) (Figure 6(a)). In DEFs, stimulation of poly (I:C) significantly activated duIFN-*β* promoter, while overexpression of VP16-His blocked the IFN-*β* promoter's activity, indicating that the presence of different small tags in the VP16 fusion proteins did not influence the inhibitory activity of VP16. At the same time, VP16 (aa1-200) and VP16 (aa1-407) proved to have the most effective inhibitory effect in IFN-*β* promoter, so that the functional region may retain within N-terminal. Intriguingly, our current results showed that the inhibitory effect of VP16 (aa1-390), VP16 (aa110-407), and VP16 (aa110-475) slightly weakened. Moreover, comparing VP16 (aa1-200) with VP16 (aa110-475) suggested that VP16 (aa1-200) is the most responsible truncated protein for inhibiting the IFN-*β* pathway. This phenomenon might relate to the transcription domain of VP16, howbeit we have not affirmed where the transcription domain is within the VP16 of DEV.

## 4. Discussion

Various cell types can detect exogenous DNA like viral and synthetic dsDNA. In recent years, a growing number of cellular DNA sensors have been identified [37, 38, 80, 81], such as AIM2 (absent in melanoma 2), RNA polymerase III [82], IFI16 (IFN-*γ*-inducible protein 16) [83], cGAS (cyclic GMP-AMP synthase), and DAI/ZBP1 (DNA-dependent activator of IRFs/Z-DNA binding protein 1) [84]. Among these, cGAS has been considered to be a major cytosolic DNA sensor responding to DNA virus infection that induces an interferon response [77]. Albeit another nuclear DNA sensor IFI16 can also sense *α*-herpesvirus DNA in a way different from cGAS, followed by cellular innate immune responses, IFI16 is dispensable in activating the downstream STING/TBK-1/IRF3 pathway where cGAS playing an indispensable role is required in this signaling cascade [85]. In the immune system of poultry, RIG-1 (retinoic acid-inducible gene I) and IRF3 (IFN regulatory factor 3) are missing [86]. Interestingly, recent research concerning chicken IRF7 indicated that IRF7 took place of IRF3 in the cGAS-STING pathway [69]. Considering that HSV-1 VP16 plays an important role in immune suppression by binding to IRF3 and physically associating with CBP to dampen host antiviral signaling, therefore we venture to guess that DEV VP16 may suppress IFN-*β* activation at the IRF7 level [87]. In this research, our results reveal a novel role for DEV VP16 that it can overtly inhibit the IFN-*β* mRNA transcription induced by poly (I:C) or poly (dA:dT) and also dampen IFN-*β* promoter activity in DEF by directly binding to IRF7 followed by interruption of downstream antiviral signaling activation, thereby helping DEV retreat from IFN-*β*-mediated defense.

Duck enteritis virus is the kind of classic double-stranded DNA virus whose infections are usually lifelong and hard to eradicate. An efficient innate immune response against these viruses is critical, not only as the first line of host defense against viral infection but also for mounting more specific and robust adaptive immunity against the virus [88]. However, DNA virus has evolved multiple strategies to evade the host antiviral response for their infection and persistence. In the case of HSV-1, a well-studied *α*-herpesvirus, a repertoire of dozens of immunomodulatory viral proteins which interfere with antiviral signaling cascades has been identified, such as VP16 [87], ICP0 [89], VHS [90], Us11 [91], and ICP27 [92]. Among the immune evasion proteins of HSV-1, VP22 [93], VP24 [94], ICP27, VP11/12 [95], and UL24 [96] specially abrogate the type I IFN production through the cGAS-STING DNA sensing pathway. In the present study, we discovered that DEV is unable to stimulate strong type I IFN responses in vitro. At the same time, WT DEV stored in our laboratory could obviously suppress IFN-*β* promoter activity through the cGAS-STING pathway. Nevertheless, no research has reported whether the proteins of DEV could hinder type I IFN production. Here, in this research, for the first time we discovered that DEV tegument protein VP16 has a strong capacity to dampen the IFN-*β* activation through the cGAS-STING pathway. This tegument protein may take part in the lifelong chronic latent infection of ducks who are suffering from DEV.

IRF7 (IFN regulatory factor 7) highly homologous with IRF3 is strongly induced by type I IFN-mediated signaling, acting as one of the key transcription factors to induce type I IFN in a positive feedback loop [97]. TBK1 (TANK Binding Kinase 1) phosphorylates IRF7 in the cytoplasm, forming a homodimer which migrates into the nucleus where it activates the IFN-*β* promoter [98] (Figure 4). Therefore, a great variety of viral proteins targeting IRF7 directly or indirectly help viruses to hinder the host antiviral activity. KSHV (Kaposi's sarcoma-associated herpesvirus) ORF45, for example, blocks phosphorylation and nuclear accumulation of IRF7 during viral infection [99]; EBV (Epstein-Barr virus) LF2, a tegument protein, interacts with the central inhibitory association domain of IRF7 to inhibit the dimerization of IRF7 [100]; HSV-1 (Herpes Simplex Virus type 1) ICP0 ring finger domain inhibits phosphorylation of IRF7 by targeting TBK1 and IKK*ε* [89]. MDV (Marek's disease virus) VP23 interacts with IRF7 competitively, disrupting the association between TBK1 and IRF7 [101]. In this study, we determined a novel role of DEV VP16 that it interacted with duck IRF7 but did not bind with IRF1, whereby dampening the following host antiviral activity.

In summary, for the first time, we demonstrate that DEV could succeed in escaping the cGAS-STNG-IRF7 signaling pathway in support of their long-time infection. What we can conclude from this study is that DEV VP16 encoded by the UL48 gene is an efficient immunomodulatory viral protein, especially acting as an IFN-*β* inhibitor. DEV VP16 counteracted cGAS-STING-mediated DNA sensing cascade and interacted with duck IRF7 rather than with duck IRF1 in vitro (Figure 4). Furthermore, a N-terminal domain (aa 1-200) of VP16 played an important role in inhibiting IFN-*β* promoter activity. Similarly, overexpression of truncated VP16 lacking N-terminal domain (aa 1-110) exerted a much more inferior inhibitory function compared to the one lacking C-terminal domain. We could have rescued a DEV VP16-deficient recombinant virus using BAC engineer through the deletion of the whole VP16 protein, but it was inefficient for this recombinant virus to replicate in cells. Its constricted replication feature consisted with a hypothesis that VP16 is essential for DEV, serving as an immune evasion factor, so that the pathogen can blend into the cellular environment. Mapping these host-virus interactions at a molecular level can provide clues for us to understand changes in species specificity and virulence of emerging pathogens and aid in the design of vaccine vectors. However, further investigations will be needed to explore if and how other counterparts of VP16 within DEV take part in immune evasion strategies.

## Figures and Tables

**Table 1:** Primers used in this research.

Primers	Forward (5′ ⟶ 3′)	Reverse (5′ ⟶ 3′)
pCAGGS-UL48-HA	CATCATTTTGGCAAAGAATTCGCCACCATGGATACATTTGATGAACT	TACGCCAAGCTTGGGCTGCAGCTAAGCGTAATCTGGAACATCGTATGGGTAATTATCTGGCGAGAACAACG
pCAGGS-duIRF1-Flag	CATCATTTTGGCAAAGAATTCGCCACCATGCCCGTCTCCAGAATGCG	TACGCCAAGCTTGGGCTGCAGCTACTTATCGTCGTCATCCTTGTAATCTTACAAGCCACAGGAGATGG
duIFN-*β* (rt-qPCR)	TCTACAGAGCCTTGCCTGCAT	TGTCGGTGTCCAAAAGGATGT
duMx (rt-qPCR)	TGCTGTCCTTCATGACTTCG	GCTTTGCTGAGCCGATTAAC
duOASL (rt-qPCR)	TCTTCCTCAGCTGCTTCTCC	ACTTCGATGGACTCGCTGTT
du*β*-actin (rt-qPCR)	GATCACAGCCCTGGCACC	CGGATTCATCATACTCCTGCTT
duIFN-*β* (rt-qPCR)	TCTACAGAGCCTTGCCTGCAT	TGTCGGTGTCCAAAAGGATGT
duMx (rt-qPCR)	TGCTGTCCTTCATGACTTCG	GCTTTGCTGAGCCGATTAAC
duOASL (rt-qPCR)	TCTTCCTCAGCTGCTTCTCC	ACTTCGATGGACTCGCTGTT

## Data Availability

The data used to support the findings of this study are available from the corresponding authors upon request.
